# Waning of IgG, Total and Neutralizing Antibodies 6 Months Post-Vaccination with BNT162b2 in Healthcare Workers

**DOI:** 10.3390/vaccines9101092

**Published:** 2021-09-28

**Authors:** Jean-Louis Bayart, Jonathan Douxfils, Constant Gillot, Clara David, François Mullier, Marc Elsen, Christine Eucher, Sandrine Van Eeckhoudt, Tatiana Roy, Vincent Gerin, Grégoire Wieers, Christine Laurent, Mélanie Closset, Jean-Michel Dogné, Julien Favresse

**Affiliations:** 1 Department of Laboratory Medicine, Clinique St-Pierre, 1340 Ottignies, Belgium; jean-louis.bayart@cspo.be (J.-L.B.); tatiana.roy@cspo.be (T.R.); vincent.gerin@cspo.be (V.G.); 2 Department of Pharmacy, Namur Research Institute for LIfe Sciences, University of Namur, 5000 Namur, Belgium; constant.gillot@unamur.be (C.G.); jean-michel.dogne@unamur.be (J.-M.D.); julien.favresse@slbo.be (J.F.); 3 Qualiblood s.a., 5000 Namur, Belgium; clara.david@qualiblood.eu; 4 Department of Laboratory Medicine, Université Catholique de Louvain, 5530 Yvoir, Belgium; francois.mullier@uclouvain.be (F.M.); melanie.closset@uclouvain.be (M.C.); 5 Department of Laboratory Medicine, Clinique St-Luc Bouge, 5004 Bouge, Belgium; marc.elsen@slbo.be (M.E.); c.eucher@labstluc.be (C.E.); 6 Department of Internal Medicine, Clinique St-Luc Bouge, 5004 Bouge, Belgium; sandrine.vaneeckhoudt@slbo.be; 7 Department of Internal Medicine, Clinique St-Pierre, 1340 Ottignies, Belgium; gregoire.wieers@cspo.be; 8 Department of Internal Medicine, Université Catholique de Louvain, CHU UCL Namur, 5530 Yvoir, Belgium; christine.laurent@uclouvain.be

**Keywords:** COVID-19, SARS-CoV-2, mRNA vaccine, BNT162b2, antibody response, neutralizing antibodies

## Abstract

Data about the long-term duration of antibodies after SARS-CoV-2 vaccination are still scarce and are important to design vaccination strategies. In this study, 231 healthcare professionals received the two-dose regimen of BNT162b2. Of these, 158 were seronegative and 73 were seropositive at baseline. Samples were collected at several time points. The neutralizing antibodies (NAbs) and antibodies against the nucleocapsid and the spike protein of SARS-CoV-2 were measured. At day 180, a significant antibody decline was observed in seronegative (−55.4% with total antibody assay; −89.6% with IgG assay) and seropositive individuals (−74.8% with total antibody assay; −79.4% with IgG assay). The estimated half-life of IgG from the peak humoral response was 21 days (95% CI: 13–65) in seronegative and 53 days (95% CI: 40–79) in seropositive individuals. The estimated half-life of total antibodies was longer and ranged from 68 days (95% CI: 54–90) to 114 days (95% CI: 87–167) in seropositive and seronegative individuals, respectively. The decline of NAbs was more pronounced (−98.6%) and around 45% of the subjects tested were negative at day 180. Whether this decrease correlates with an equivalent drop in the clinical effectiveness against the virus would require appropriate clinical studies.

## 1. Introduction

The efficacy and safety of the two-dose regimen of BNT162b2 mRNA COVID-19 vaccine (Pfizer-BioNTech, Mainz, Germany) have been proven and led to an emergency use authorization (EUA) delivered on the 11th of December 2020 [1]. On the 23rd of August 2021, the BNT162b2 vaccine became the first COVID-19 vaccine approved by the U.S. Food and Drug Administration (FDA) [2]. Real-world data on the BNT162b2 also confirmed the high effectiveness of this vaccine in reducing laboratory-confirmed infection and viral load in infected individuals, as well as reducing COVID-19 hospitalization and death [3,4,5,6,7].

Around the world, massive vaccination campaigns started in early 2021 and data about the immunological response emerged in the literature to document the evolution of humoral response in subjects vaccinated against SARS-CoV-2 [8,9,10,11,12,13,14,15]. In most seronegative subjects, antibody response was positive two weeks after the first dose and the peak response was observed around 28 days [13,16]. The antibody titers were also higher in previously infected individuals compared to seronegative subjects and recent studies found a decline in antibody titers at 3 months [13,17,18,19]. This antibody decline needs to be well monitored because it may provide important information to support the decision-making on the potential use of a booster dose. However, longer-term kinetics data of the humoral response after the two-dose regimen of BNT162b2 are still scarce in the literature.

The CRO-VAX-HCP study is an ongoing multicenter, prospective, and interventional study designed to assess the antibody response in a population of healthcare professionals (HCPs) having received two doses of the BNT162b2 mRNA COVID-19 vaccine. We report here an interim analysis on the data obtained on the humoral response after a 6-month follow-up.

## 2. Materials and Methods

### 2.1. Study Design and Participants

The CRO-VAX HCP study is an ongoing multicenter, prospective, and interventional study designed to assess the antibody response in a population of HCPs having received two doses of the BNT162b2 mRNA COVID-19 vaccine (Comirnaty^®^), as described in Supplementary Table S1. The study was approved by a central ethical committee (approval number: 2020-006149-21) and a total of 231 participants were enrolled in the study. All participants provided informed consent prior to data and specimen collections. Participants received the first vaccine dose from 18 January 2021 to 17 February 2021. The second dose was administered 21 days after the first one. All volunteers underwent blood drawn within 2 days before the first vaccine dose. Samples were then collected after 14, 28, 42, 56, 90 and 180 days following the first dose. Blood samplings performed earlier or later than the expected blood collection times were allowed with a maximal allowed percentage of 10% (i.e., 180 days ± 18 days). Subjects having levels of anti-NCP and anti-S antibodies at baseline above the positivity cut-offs of the assays were considered seropositive while the others are considered COVID-19 naïve and are classified as seronegative.

### 2.2. Analytical Procedures

Antibodies against the receptor binding domain (RBD) of the S1 subunit of the spike protein were measured at each time point by two different analytical methods: the Elecsys^®^ assay that measured SARS-CoV-2 total antibodies (combination of IgG, IgM and IgA) (Roche Diagnostics, Machelen, Belgium) with a positivity cut-off of 0.8 U/mL and the Architect^®^ assay that measured SARS-CoV-2 IgG (Abbott, Wavre, Belgium) with a positivity cut-off of 50 arbitrary unit (AU)/mL. Total antibodies against the SARS-CoV-2 nucleocapsid (anti-NCP; Roche Diagnostics, Machelen, Belgium) were also measured. Results above 0.165 COI (cut-off index as reported previously) [20] were considered positive. For the total antibody assay, alternative cut-offs (i.e., 15 U/mL and 133 U/mL) [21,22] were also investigated.

Neutralizing antibodies (NAbs) were assessed on a subset of 60 subjects at different time points (0, 28, 90 and 180 days) using a pseudo-virus neutralization test (pVNT) as described elsewhere [23,24]. For this latter assay, samples were considered negative if the half-maximal inhibitory concentration (IC_50_) value was below the dilution of 1/20.

### 2.3. Statistical Analyses

Means and 95% confidence intervals (95% CI) were used for data description. The between-group differences of antibody titers were tested using a Tukey multiple comparison test. A multiple testing correction was applied in the multiple group comparison. For kinetics modeling, a one compartment modeling was used to describe the kinetics of the antibody response in seropositive and seronegative subjects, assuming a steady decay rate over time. The half-life (T_1/2_) was obtained from the one compartment modeling which permitted the calculation of the elimination rate of the antibodies. The mean time needed to cross the various thresholds of interest defined above was also determined based on this model. Correlation studies (IgG, total antibodies and NAbs) were evaluated with a Spearman’s rank correlation test. A Cohen’s kappa agreement test was also performed between assays. Statistical analyses were performed using GraphPad Prism 9.0.1 (GraphPad Software, San Diego, CA, USA, www.graphpad.com, accessed on 22 September 2021) and JMP Pro 16.0.0 (JMP®, Version 16.0.0. SAS Institute Inc., Cary, NC, USA, 1989–2021). A *p*-value < 0.05 was considered significant.

## 3. Results

### 3.1. Demographic Data

Among the participants, 170 (73.6%) were female (mean age = 43 years; range, 23–66 years) and 61 (26.4%) were male (mean age = 43 years; range, 23–64 years). A total of 158 subjects (68.3%) were COVID-19 naïve and were categorized as seronegative at baseline while 73 (31.6%) were seropositive. All demographic data of the population are presented in Supplementary Table S1.

### 3.2. Anti-NCP Antibodies

Among the cohort, anti-NCP antibodies remained stable in seropositive participants up to 6 months compared to pre-vaccinal titers (*p* > 0.05) (Supplementary Figure S1). At the individual level, 3 participants (1.3%) had a significant increase in their anti-NCP antibody levels. The first subject was seronegative before vaccination and had a positive RT-PCR 93 days after the first vaccine dose. The B.1.1.7 variant was identified by sequencing. The subject had a close contact with an infected patient and was asymptomatic. The second subject was seropositive at baseline with a documented positive RT-PCR in April 2020 and reported a positive RT-PCR 71 days after the first dose. The subject developed minor symptoms. No sequencing was available. The third subject was seronegative before vaccination and reports a positive RT-PCR carried out in the context of persisting flu-like symptoms, only 2 days after the first dose. All the subjects with symptoms recovered without sequelae.

### 3.3. IgG and Total Antibodies

**Figure 1:**
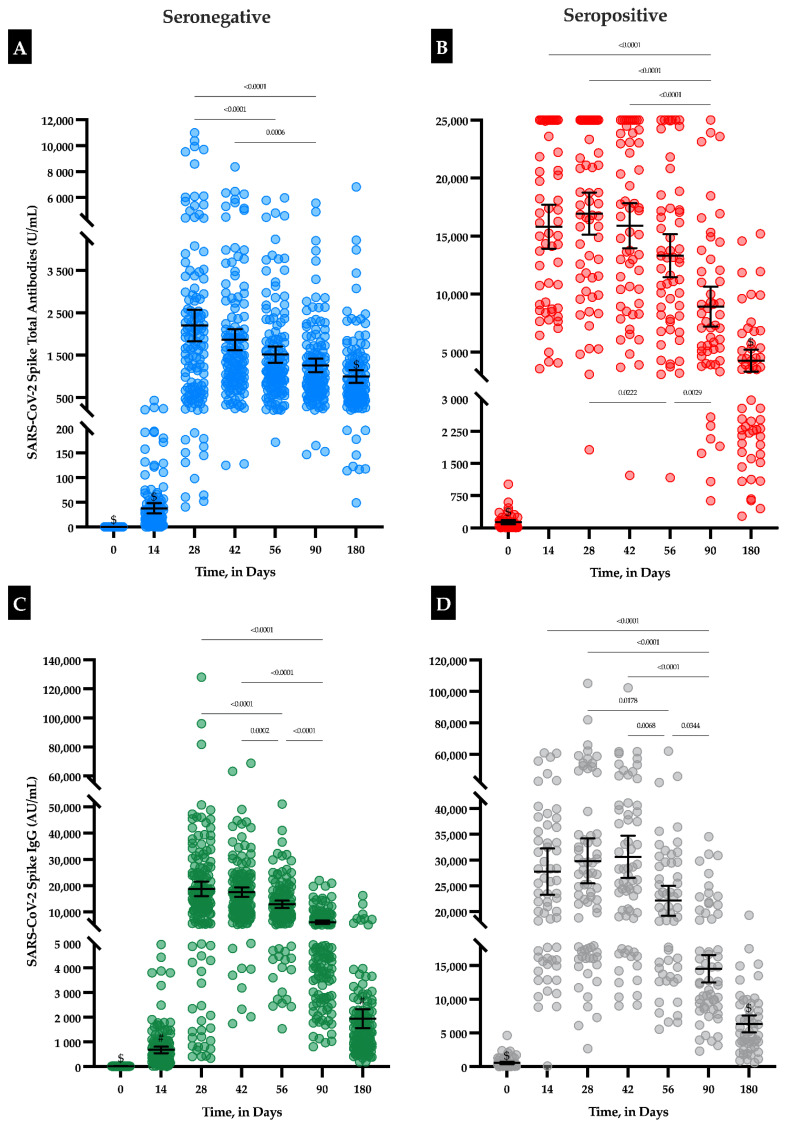
(Previous page). Evolution of SARS-CoV-2 spike antibodies (U/mL) in seronegative (A and C for total antibodies and IgG, respectively) and seropositive individuals (B and D for total antibodies and IgG, respectively) according to the time since the first vaccine dose administration. Means with 95% confidence intervals are shown. (**A**,**B**) Using the total antibody assay, an automatic dilution of 1/100 at >250 U/mL was performed by the analyzer to extend the measurement domain up to 25,000 U/mL. A total of 46 samples were rounded to 25,000 U/mL out of 1337 (3.4%). Results < 0.4 U/mL (limit of quantification) were rounded to 0.4. (**C**,**D**) Using the IgG assay, an automatic dilution of 1/4 at > 40,000 AU/mL was manually performed to extend the measurement domain to 160,000 AU/mL. Results < 21 AU/mL (limit of quantification) were rounded to 21. $, statistically different from all other groups (i.e., *p* < 0.0001); #, statistically different from all other groups (i.e., *p* < 0.0001) except between time points 14 and 180.

In seronegative individuals, the maximal antibody response was reached at day 28 with a mean total antibody titer of 2204 U/mL (95% CI: 1833–2575) and a mean IgG titer of 18,785 AU/mL (95% CI: 16,020–21,549). A continuous decrease was observed up to day 180 with an observed mean total antibody titer of 998 U/mL (95% CI: 848–1148) and an observed mean IgG titer of 1949 AU/mL (95% CI: 1565–2332), which represent a decrease of 54.7% and 89.6%, respectively (Table 1, Figure 1). In seropositive individuals, the maximal antibody response was reached at day 28 and day 42 for total and IgG antibodies, respectively. The mean total antibody titer was 16,935 U/mL (95% CI: 15,112–18,759) and the mean IgG titer was 30,678 AU/mL (95% CI: 26,600–34,755). A continuous decline was also observed between day 28 or day 42 and day 180 with a total antibody titer of 4270 U/mL (95% CI: 3324–5215), which represents a decrease of 74.8% and 79.4%, respectively (Table 1, Figure 1). All participants still had detectable anti-S antibodies 6 months after the first vaccine dose (i.e., total antibody titer ≥ 0.8 U/mL and IgG titer ≥ 50 AU/mL).

Considering each time point separately, anti-S titers of seropositive individuals were always statistically higher compared to seronegative individuals (*p* < 0.0001), except for IgG at day 180 time point (Table 1). The difference of titers between seronegative and seropositive individuals was higher when measuring total antibodies compared to IgG, but the difference tended to decrease over time (Table 1, Figure 1).

**Figure 2:**
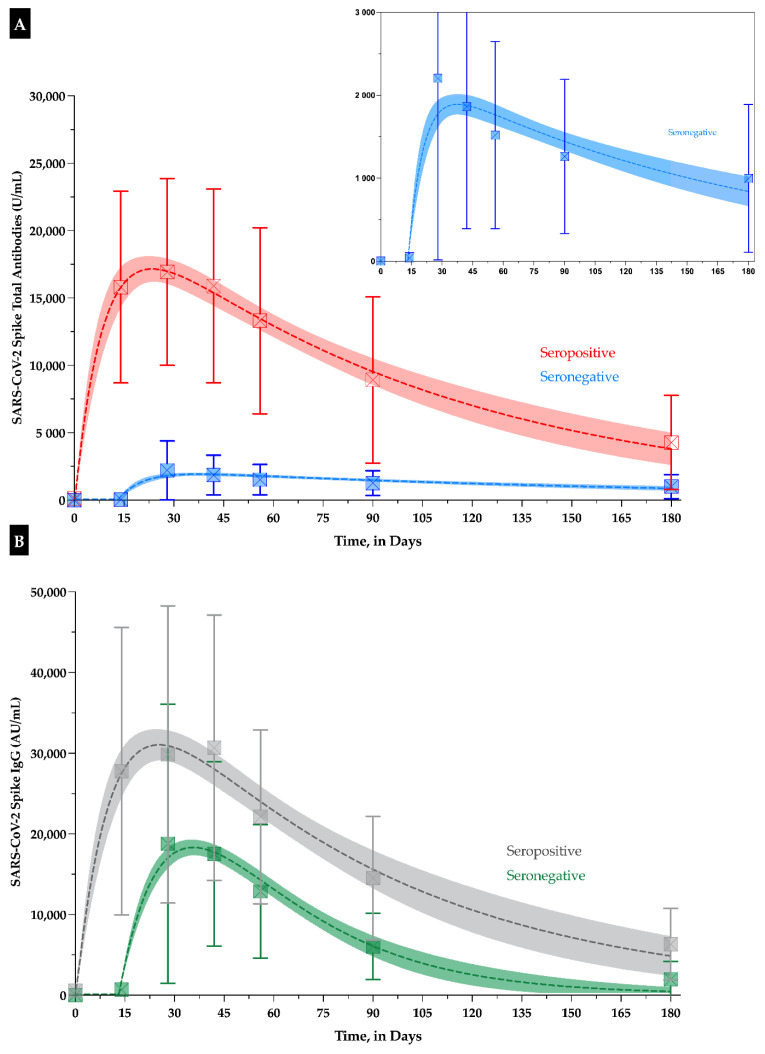
(Previous page). Kinetics models of (**A**) total antibodies and (**B**) IgG serological response. A zoom of the seronegative population is presented in the right-upper part of the Figure A. Means plus/minus standard deviation are shown at the different timepoints. The magnitude of the response depends on the analytical kit and the difference between COVID-19 naïve and seropositive individuals is less marked with IgG than with total antibodies.

Using the kinetics model, the time to maximum concentration (T_MAX_) in seronegative subjects for total antibodies and IgG was comparable with 36.3 days (95% CI: 30.2–42.5) versus 34.5 days (95% CI: 31.7–37.2). The estimated T_1/2_ for total antibodies was 114 days (95% CI: 87–167) and was significantly longer than the 21 days (95% CI: 13–65) obtained for IgG. In seropositive subjects, the T_MAX_ for total antibodies and IgG were also comparable (23.3 days (95% CI: 18.7–28.0) versus 25.0 days (95% CI: 20.4–29.9)), and was shorter compared to seronegative. The estimated T_1/2_ for total antibodies was 68 days (95% CI: 54–90) and slightly longer compared to IgG (i.e., 53 days, 95% CI: 40–79) (Figure 2).

According to the model, a mean time of 229 days (95% CI: 134–277) in seronegative and 529 days (95% CI: 283–623) in seropositive would be needed to cross the threshold of 50 AU/mL for the IgG assay. For the total antibody assay, a mean time of 830 days (95% CI: 508–1000) in seronegative and 718 days (95% CI: 425–826) in seropositive would be needed to cross the threshold of 15 U/mL, which was defined by the manufacturer as a cut-off for detection of inhibitory effects [21]. Using the threshold of 133 U/mL [22], the mean time needed would be 470 days (95% CI: 341–585) and 507 days (95% CI: 359–591) in seronegative and seropositive subjects, respectively. 

Among the 1443 samples analyzed on both assays, IgG and total anti-S antibodies showed an almost perfect agreement (i.e., Cohen’s kappa = 0.97) with a Spearman’s correlation coefficient of 0.892 (95% CI: 0.881–0.902; *p* < 0.0001) (Supplemental Figure S2). 

### 3.4. Neutralizing Antibodies

**Figure 3:**
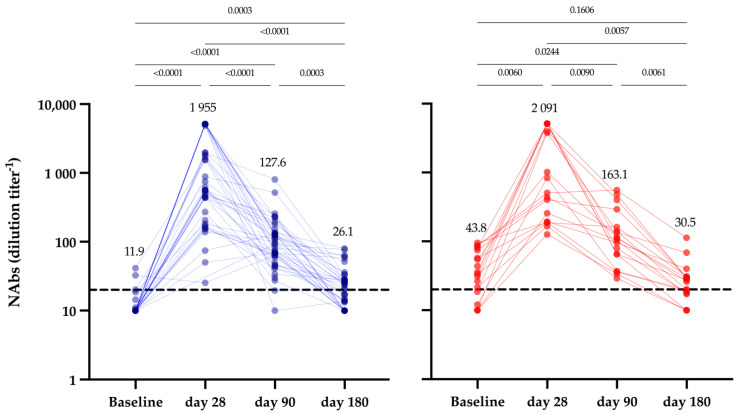
Evolution of SARS-CoV-2 NAbs in seronegative (blue, *n* = 42) and seropositive individuals (red, *n* = 18) at baseline and 1 month, 3 months and 6 months after the first vaccine shot.

In the 42 seronegative subjects included in this subgroup analysis, NAbs increased from a mean dilution factor of 11.9 (95% CI: 9.96–13.8) at day 0 to 1955 (95% CI: 1287–2622) at day 28, which represents an increase of 99.4% (*p* < 0.0001) with all subjects having detectable NAbs at day 28. At day 90 and day 180, the mean dilution factors decreased to 127.6 (95% CI: 84.3–170.9) and 26.1 (95% CI: 20.1–32.1), which represents, respectively, a significant decrease of 93.5% and 98.7% compared to day 28. At these time points, the positivity rates dropped at 95.2% and 45.0% (Figure 3), respectively. In the 18 seropositive subjects, 72.2% of the subjects had detectable NAbs at baseline. At day 28, the mean dilution factor increased from 43.8 to 2091, which represents an increase of 97.9% (*p* < 0.001). At day 90 and day 180, the mean dilution factors were 163.1 (95% CI: 83.5–242.6) and 30.5 (95% CI: 18.2–42.7), which represents a significant decrease of 92.2% and 98.5%, respectively. All subjects had detectable levels of NAbs at day 28 and day 90 but the positivity rate decreased to 44.4% at day 180. Considering each time point separately, NAbs of seropositive individuals were not statistically different compared to seronegative individuals (*p* > 0.9998). The kinetics model found that the estimated T_1/2_ of NAbs in this subgroup was 16 days (95% CI: 9 to 59 days) and that the time to reach the negativity cut-off was 135 days (95% CI: 55–179 days). A significant correlation with total antibodies was found (r = 0.63, *p* < 0.0001) (Supplemental Figure S3) but the strength of agreement was moderate with the manufacturer’s cut-off (Cohen’s kappa = 0.60). The use of alternative cut-offs (15 and 133 U/mL) did not increase the agreement (0.58 and 0.54, respectively). For the IgG assay, we observed a better correlation (r = 0.78, *p* < 0.0001), still with a moderate strength of agreement (Cohen’s kappa = 0.66) (Supplemental Figure S3). No alternative cut-off could enhance the observed agreement.

## 4. Discussion

In this study, compared to the peak antibody response, a significant antibody decline 6 months post-vaccination with BNT162b2 COVID-19 vaccine was reported. The decline was highly significant for total antibodies, IgG and NAbs in both seronegative and seropositive participants.

In COVID-19 naïve subjects, the decline of total antibodies at 6 months was lower (i.e., −54.7%) than the decline of IgG (i.e., −89.6%) or NAbs (−98.7%), while in seropositive participants, the decline of total antibodies and IgG at 6 months was quite similar (−74.8% versus −79.4%) and lower than the decline of NAbs (−98.7%). The distinct kinetics observed for the total antibody assays compared to IgG may be explained by the additional response of non-IgG antibody isotypes, which may persist several month post-vaccination [25,26]. On the other hand, in subjects who had already developed an immune response due to exposure to SARS-CoV-2 or occurrence of COVID-19 disease, the serological response following a de novo exposure to the antigen is mainly dominated by the IgG response while the IgM response is reduced, or even absent. This may explain why the total and IgG antibodies declines are closer in the seropositive subgroup [27,28]. Interestingly, in seropositive subjects, the anti-NCP antibodies level did not significantly decline over at least day 180 [29]. Some groups have suggested that anti-NCP antibodies confer additional immunity in seropositive subjects but this is subject to debate in the literature [30]. Besides, in our subgroup analysis, we did not identify a difference in terms of NAb titers between seropositive and seronegative subjects (Table 1).

Data about the long-term antibody kinetics in BNT162b2 recipients are still scarce. In this study, we reported antibody T_1/2_ ranging from 21 to 114 days depending on the type of antibody testing and the baseline serological status of the participants. Such an important elimination rate implies that those antibodies are certainly only produced briefly at the time of vaccination and heightened by an anamnestic response in seropositive subjects. 

Israel et al. recently found that the mean SARS-CoV-2 IgG antibody titer (Abbott Architect^®^) after BNT162b2 vaccination decreased by 93.7% at 6 months (i.e., 765 AU/mL) compared to the highest mean antibody response (i.e., 12,153 AU/mL) [31]. These data are confirmed by our study, especially in the seronegative cohort. The higher mean titers observed in our study may be related to the lower age of our cohort compared to the one of Israel et al. (42.0 versus 56.5 years), which has been reported as a confounding factor for antibody response [32,33]. Moreover, the antibody decline was also higher compared to convalescent patients where a drop of 4 to 7% every month has been reported compared to the drop observed in our vaccinated subjects [31,34]. Finally, the proportion of vaccinees below the threshold of 50 AU/mL was higher (i.e., 16.1% at 6 months) compared to the one in convalescent subjects (i.e., 10.8% at 9 months) [31]. In our study, no participant had IgG or total antibody titers below the positivity threshold (i.e., 50 AU/mL). In contrast to Israel et al., we also enrolled subjects categorized as seropositive before the first vaccine injection and observed an IgG decrease of 79.4% in this group (Figure 1). As reported elsewhere, the vaccination with BNT162b2 elicited much higher total antibody and IgG titers compared to ones obtained in convalescent patients [12,26,31]. In our model, the T_MAX_ for total antibodies and IgG was reached at a similar timeframe in the two cohorts. The model also predicts a drop of IgG below the positivity threshold after 229 days (95% CI: 134–277 days) for seronegative and after 529 days (95% CI: 283–623 days) for seropositive individuals. Regarding total antibodies, depending on the cut-off used, these times range from 470 (95% CI: 341–585 days) to 830 days (95% CI: 508–1000 days) in seronegative and from 507 (95% CI: 359–591 days) to 718 days (95% CI: 42–826) in seropositive subjects. These predictions need to be confirmed by subsequent sampling times to refine the reliability of the model. However, these data could already support the different government and competent authorities in the decisions that will need to be taken for the next steps of the vaccination strategy [35]. These observations are in line with the statement of the CEO of Pfizer who declared that a third vaccine dose would be likely needed 12 months following the first shot [2]. If assuming that the antibody decline is constant over time, our kinetics model predicts a decrease below the positivity threshold between 229 and 830 days, depending on the assay used (total versus IgG antibodies) and the serological status of the subject before vaccination. The aim will be to keep an effective humoral response to protect vaccinated subjects against the wild-type SARS-CoV-2, but, more importantly, against the variants of concern. It is therefore important to have reliable models to predict when the drop will be too high to maintain a sufficient humoral response. In addition, as some variants of concern have demonstrated an immune escape, it is important to realize that our models may even be optimistic since higher NAb titers may be needed to provide a similar degree of protection than the one reported during the clinical development of these vaccines [35].

More and more data support the concept that NAbs correlate with protection against infection and it has been suggested that it may serve in the future as a biomarker that can ensure a proper protection at the individual level [36]. Earle et al. observed a relatively good correlation between the neutralizing (r_s_ = 0.79) or binding antibodies (r_s_ = 0.93) and vaccine efficacy [37]. In addition, in a study on 1497 healthcare professionals having received the two-dose regimen of the BNT162b2 vaccine, Bergwerk et al. identified lower NAbs and IgG titers in the 39 subjects that developed a SARS-CoV-2 infection despite a full-vaccination scheme. This supports the concept that NAbs, or assays that correlate with NAbs, may be an appropriate indicator of the protection at the individual level [38]. Nevertheless, the fact that only 3 subjects became positive to SARS-CoV-2 post-vaccination is reassuring, although this may result from continued physical distancing and good hygiene.

The automated assays used in the present study (i.e., Roche Elecsys^®^ and Abbott Architect^®^) do not specially measure NAbs but a significant correlation between the anti-S assay from Roche Diagnostics (r = 0.63, *p* < 0.0001) or Abbott (r = 0.78; *p* < 0.0001) and pVNT was found (Supplemental Figure S2) [39,40]. Nevertheless, these correlations were weak and do not reflect the percentage of subjects with NAbs under the positivity threshold at 6 months, i.e., 55% with the pVNT assay vs. 0% with both total and IgG antibodies. Several other studies reported the same conclusions with various serological assays available on the market [16,40,41,42]. These discrepancies seem to suggest that the manufacturers’ cut-off is not adequate to reflect the neutralizing capacity and should be significantly increased to reach a better agreement with NAbs, although improvements seem difficult to reach (Supplementary Figure S3). Indeed, some patients presenting high levels of total or IgG antibodies at 6 months no longer have NAbs at the same time point. This highlights the fact that we are probably still far from reaching a substitution of pVNT assays by the surrogate IgG or total antibodies assays currently on the market. Thus, the clinical implications of the waning in NAbs we observed is not yet clear from a clinical point of view and the establishment of thresholds associated with protection are still needed, but the link between low NAb titer and breakthrough infection may not be excluded and may justify the application of appropriate vaccination strategies, especially in frail patients [38,43,44,45]. 

Despite the call of the WHO to temporarily halt the administration of COVID vaccine boosters [46], some countries have already decided to administer such booster doses. In Israel and France, a third dose of the BNT162b2 vaccine is given to people over 50 or 65 and to other vulnerable persons [47]. In Germany, UK and USA, boosters are planned to be administered to certain groups of persons. In an editorial published in Nature on the 17th of August 2021, authors agreed with the WHO to call a temporary halt to COVID vaccine boosters. Indeed, at the time of the editorial, 58% of people in high-income countries had received at least one vaccine dose compared to 1.3% in low-income countries. In some particular cases (i.e., immunosuppressant drugs), boosters might be warranted, but there is still little evidence that these additional shots are needed to protect fully vaccinated people [46].

Finally, besides the humoral response, there are still few data concerning the cell-mediated immunity responses and in which extent this protection contributes to the long-term efficacy of the vaccines. Indeed, there is increasing evidence that an early and coordinated T cell response is associated with less severe COVID-19 and provides longer-term protection, even against the variants of concern [48,49,50].

## 5. Conclusions

A highly significant decrease in NAbs, IgG and total antibodies in both seropositive and seronegative subjects, 6 months after the administration of the first dose of BNT162b2, was observed. The decline of NAbs was more pronounced and around 45% of the subjects tested were negative at 6 months. Further studies are needed to elucidate the relationship between the decline of the humoral response and the clinical efficacy of the vaccine. Moreover, with various kinetics observed, our results also raise the question of which antibody types will be the most clinically relevant to assess the humoral decline, since none of those reported in this study seem to show a sufficient correlation and agreement with the neutralizing capacity. This study has a planned follow-up of two years, with the next blood sampling campaign planned in January 2022. This will permit us to further refine the kinetics model and to provide a better estimate of the antibody response in both seropositive and seronegative individuals.

## Figures and Tables

**Table 1:** Evolution of SARS-CoV-2 spike antibodies (U/mL) in seronegative and seropositive persons using the Roche Elecsys^®^, the Abbott Architect^®^ assays and the pseudovirus neutralizing test. Means with 95% confidence intervals are reported. The between group difference of antibody titers were tested using a Tukey multiple comparison test. A multiple testing correction was applied in the multiple group comparison. *p*-value < 0.05 was considered significant. ^†^ pVNT have only been performed in 60 subjects.

SARS-CoV-2 Total Antibodies				
	Seronegative	Seropositive	Ratio +/−	*p*-Value
**Before first dose**	0.40 (0.39–0.41) U/mL	132.0 (86.1–177.6) U/mL	330	<0.0001
**14 days**	38.2 (27.7–48.6) U/mL	15,540 (13,606–17,473) U/mL	406	<0.0001
**28 days**	2204 (1883–2575) U/mL	16,935 (15,112–18,759) U/mL	7.7	<0.0001
**42 days**	1863 (1613–2113) U/mL	15,896 (13,968–17,824) U/mL	8.5	<0.0001
**56 days**	1517 (1326–1708) U/mL	13,315 (11,464–15,165) U/mL	8.8	<0.0001
**90 days**	1262 (1104–1420) U/mL	8919 (7201–10,637) U/mL	7.1	<0.0001
**180 days**	998 (848–1148) U/mL	4270 (3324–5215) U/mL	4.3	<0.0001
**SARS-CoV-2 IgG Antibodies**				
	**Seronegative**	**Seropositive**	**Ratio +/−**	***p*-value**
**Before first dose**	21.2 (20.8–21.6) AU/mL	556.6 (385.3–727.9) AU/mL	26.3	<0.0001
**14 days**	679.9 (548.7–811.2) AU/mL	27,753 (23,226–32,239) AU/mL	40.8	<0.0001
**28 days**	18,785 (16,020–21,549) AU/mL	29,845 (25,484–34,206) AU/mL	1.6	<0.0001
**42 days**	17,507 (15,685–19,328) AU/mL	30,678 (26,600–34,755) AU/mL	1.8	<0.0001
**56 days**	12,862 (11,441–14,284) AU/mL	22,115 (19,174–25,056) AU/mL	1.7	<0.0001
**90 days**	6050 (5371–6729) AU/mL	14,509 (12,477–16,541) AU/mL	2.4	<0.0001
**180 days**	1949 (1565–2332) AU/mL	6333 (5072–7593) AU/mL	3.2	0.342
**Pseudovirus Neutralization Test ^†^**				
	**Seronegative**	**Seropositive**	**Ratio +/−**	***p*-value**
**Before first dose**	11.9 (10.0–13.8)	43.8 (29.0–58.5)	3.7	<0.0001
**28 days**	1955 (1287–2622)	2091 (981–3202)	1.1	0.823
**90 days**	127.6 (84.3–170.9)	163.1 (83.5–243)	1.3	0.390
**180 days**	26.1 (20.1–32.1)	30.5 (18.2–42.7)	1.2	0.463

## Data Availability

The data presented in this study are available on request from the corresponding author. The data are not publicly available according to the ethical committee decision on the conduct of this study.

## Supplementary Materials

The following are available online at https://www.mdpi.com/article/10.3390/vaccines9101092/s1, Figure S1: Evolution of SARS-CoV-2 nucleocapsid antibodies (COI) in seronegative (yellow) and seropositive individuals (maroon) according to the time since the first vaccine dose administration, Figure S2: Correlation of IgG and total anti-spike antibody levels. Figure S3: Correlation of NAbs, total and IgG antibodies levels against the spike protein, Table S1: Demographic data.
