# Supplementary material for: Waning of IgG, Total and Neutralizing Antibodies 6 Months Post-Vaccination with BNT162b2 in Healthcare Workers
Source: Vaccines (Basel). 2021 Sep 28;9(10):1092. doi: 10.3390/vaccines9101092 (PMC8540417; doi:10.3390/vaccines9101092)
Supplement: Supplementary file 1 [file vaccines-09-01092-s001.zip › vaccines-1399894-supplementary.pdf]

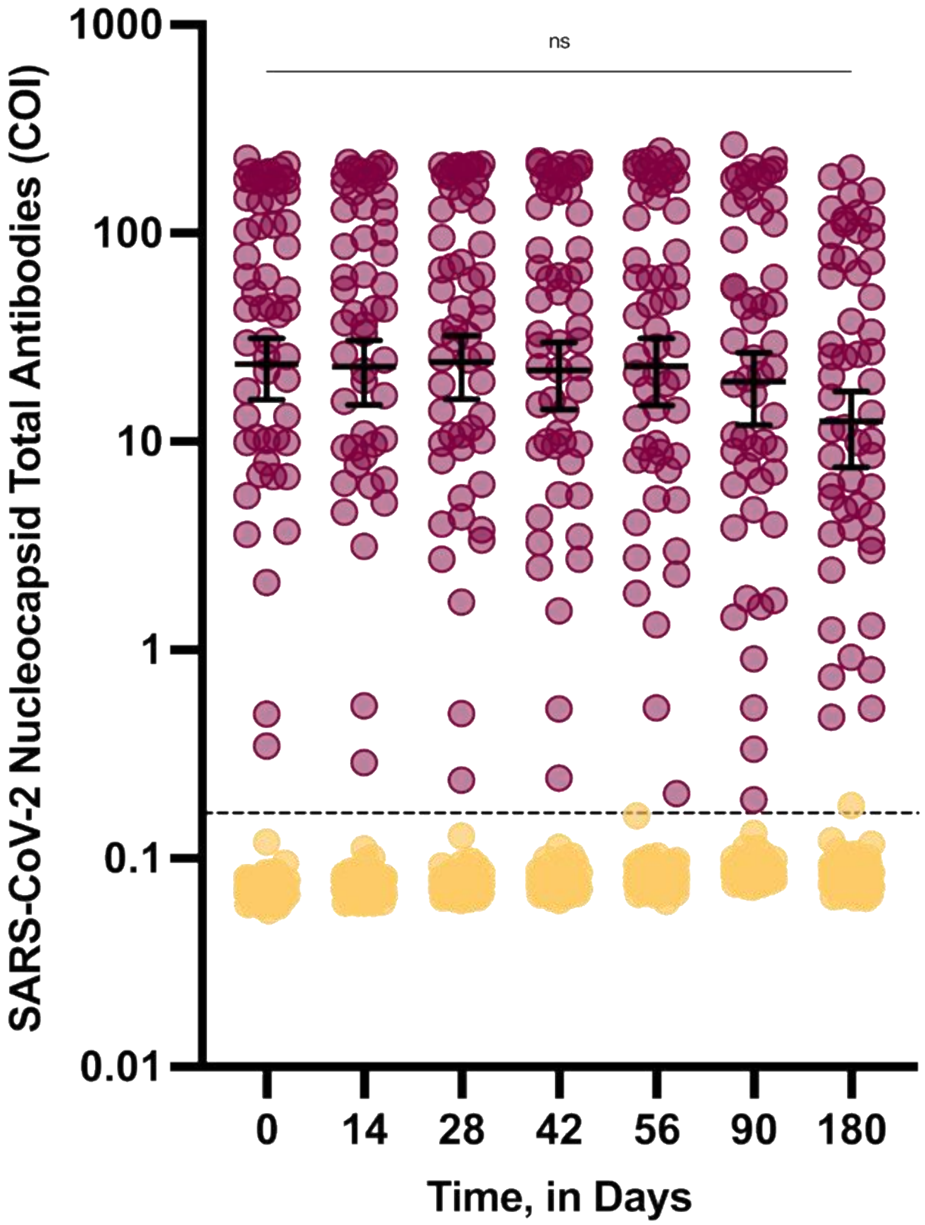

Figure S1. Evolution of SARS-CoV-2 nucleocapsid antibodies (COI) in seronegative (yellow) and seropositive individuals (maroon) according to the time since the first vaccine dose administration. Means with 95% confidence intervals (log_{10}) are shown. The black dotted line corresponds to the positivity cut-off (i.e. 0.165 COI). ns = non significant differences between timepoints ($P < 0.05$). Subjects that developped anti-NCP during the study were excluded from the analysis.

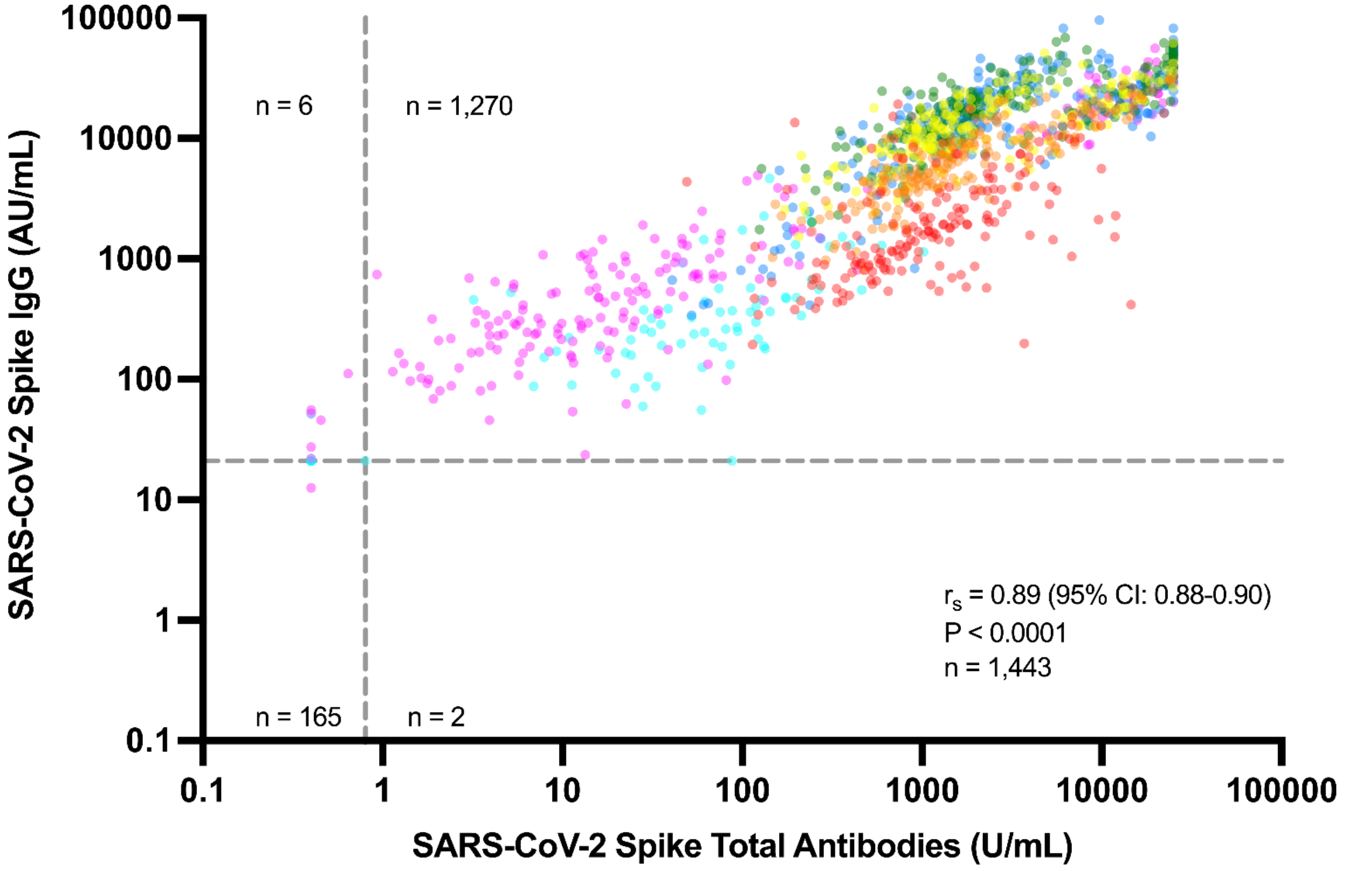

Figure S2. Correlation of IgG and total anti-spike antibody levels. Spearman's rho (r), 95% confidence interval (95% CI) , and p-value are reported in the figure. Manufacturer's cut-off for both assays are represented with doted lines. Different colors were used to discriminate the different timepoints. (165 points were negative with the two methods and are plotted as 0.8;21).

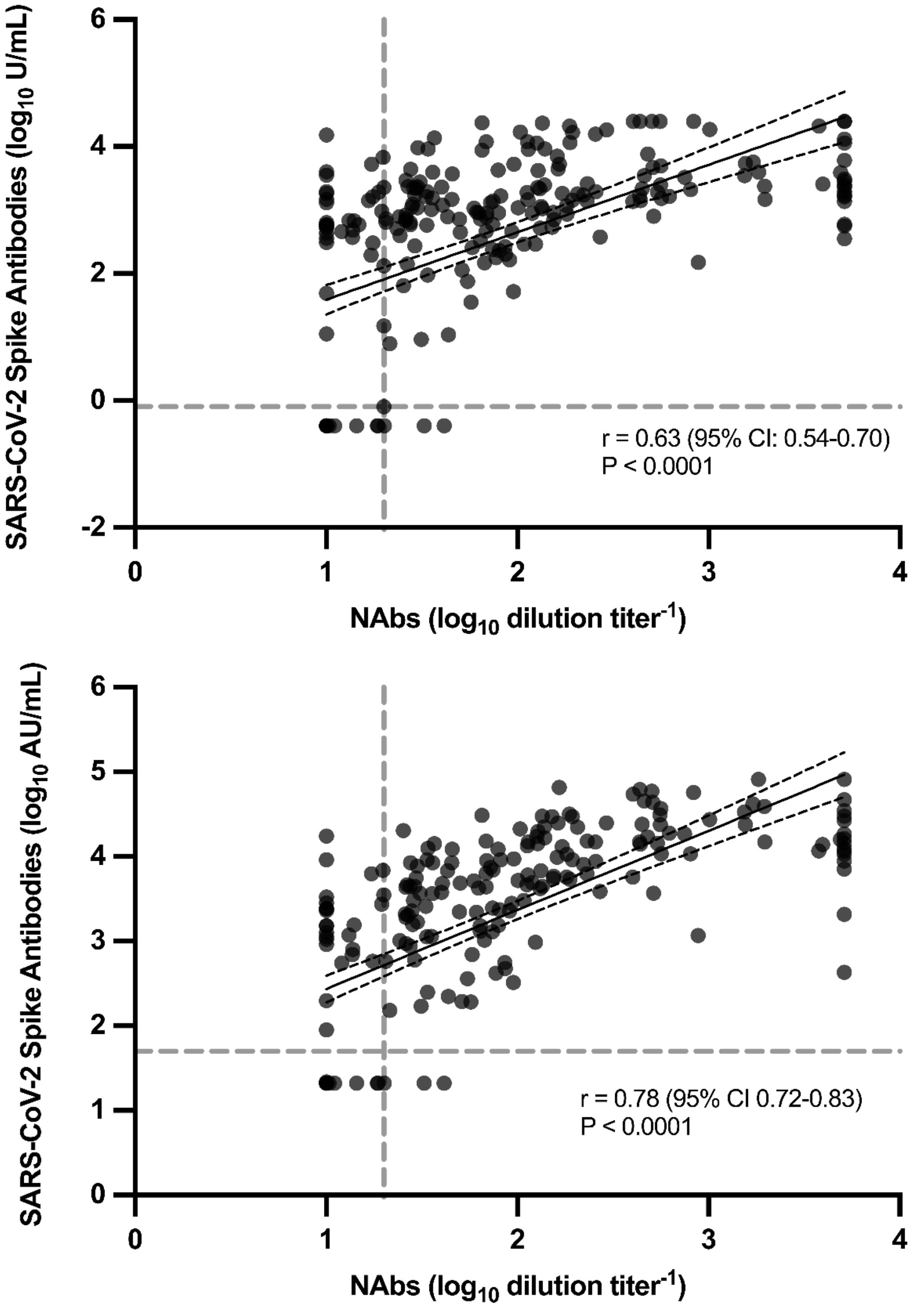

Figure S3. Correlation of NAbs, total and IgG antibodies levels against the spike protein. Spearman's rho (r), 95% confidence interval (95% CI), and p-value are provided in the figure. Manufacturer's cut-off of 0.8 U/mL for total antibodies, 50 AU/mL for IgG antibodies and positivity cut-off of 1/20 for pVNT are represented with grey doted lines.

Table S1. Demographic data. † Body mass index (BMI) data were available only in 220 subjects.

**Demographic Characteristics of the Participants (n = 231)**	
**Sex – no. (%)**	
Female	169 (73)
Male	62 (27)
**Age group – no. (%)**	
≤ 45 years of age	139 (60)
> 45 years of age	92 (40)
**Past-COVID history – no. (%)**	**73 (32)**
Female	52 (71)
Male	21 (29)
**BMI in kg/m²** (median, range)†	23.7 (15.3 – 48.2)
**ABO blood group – no. (%)**	
A	76 (33)
B	9 (4)
AB	18 (8)
O	98 (42)
Unknown	30 (13)
**Rhesus blood group system – no. (%)**	
Negative	36 (16)
Positive	159 (69)
Unknown	36 (16)
**For female gender – no. (%) :**	
**Childbearing age**	**121 (72)**
Hormonal contraception	78 (65)
No hormonal contraception	38 (31)
Unknown	5 (4)
**Menopausal**	**48 (28)**
Hormonal replacement therapy	13 (27)
No hormonal replacement therapy	30 (63)
Unknown	5 (10)
